# Supplementary material for: The ALINFA Intervention Improves Diet Quality and Nutritional Status in Children 6 to 12 Years Old
Source: Nutrients. 2023 May 18;15(10):2375. doi: 10.3390/nu15102375 (PMC10221220; doi:10.3390/nu15102375)
Supplement: Supplementary file 1 [file nutrients-15-02375-s001.zip › nutrients-2349217-supplementary.pdf]

Supplementary Information S1: Detailed description of the ALINFA nutritional intervention

The ALINFA intervention consisted of a diet designed by the dietitians-nutritionists of the University of Navarra specially for children based on the inclusion of the different products, ready-to-eat meals and healthy recipes designed in the ALINFA consortium. Specifically, the ALINFA diet included ready-to-eat dishes (produced by Irigoyen Comedor Saludable S.L.), healthy recipes to develop at home (designed by CNTA) and products developed by the study companies as part of the project within the framework of a balanced diet.

The ALINFA diet consisted of a fixed full-day meal plan, divided into 5 meals, with information on culinary technology, ingredients and quantities, that made up a 2-week menu. The distribution of macronutrients in the diet, respect to the total energy value was: a) carbohydrates: > 50%; b) fat: < 35% and c) protein: 15-20%. Two diets with different portion sizes were established for each age group (6-9 years and 10-12 years). The lunch meal consisted on two courses and dinner was designed as a single full course. Both meals were accompanied by a dessert, yogurt or fruit, and a piece of whole wheat bread.

Table S1: ALINFA diet (10-12 years): 2-week menu

Week 1	DAY 1	DAY 2	DAY 3	DAY 4	DAY 5	DAY 6	DAY 7
**BREAKFAST**	1 glass of whole milk (200 ml) with 1 tablespoon of soluble cocoa (10 g)<br>Breakfast cereals (30g)<br>Orange (150g)	1 glass of whole milk (200 ml) with 1 tablespoon of soluble cocoa (10 g)<br>1 whole wheat toast (30 g)<br>Banana (80g)	1 glass of whole milk (200 ml) with 1 tablespoon of soluble cocoa (10 g)<br>Breakfast cereals (30g)<br>Apple (150g)	1 glass of whole milk (200 ml) with 1 tablespoon of soluble cocoa (10 g)<br>1 whole wheat toast (30 g)<br>Tangerine (150g)	1 glass of whole milk (200 ml) with 1 tablespoon of soluble cocoa (10 g)<br>Breakfast cereals (30g)<br>Kiwi (150g)	1 glass of whole milk (200 ml) with 1 tablespoon of soluble cocoa (10 g)<br>1 whole wheat toast (30 g)<br>Orange (150g)	1 glass of whole milk (200 ml) with 1 tablespoon of soluble cocoa (10 g)<br>Breakfast cereals (30g)<br>Pear (150g)
**MID-MORNING SNACK**	Snack: Rice, corn and peas with olive oil (1 portion)<br>1 handful of pistachios (15 g)	Tangerine (150g)<br>1 handful of walnuts (15 g)	1 small sandwich of whole wheat bread (45 g) with ham (40 g)	Banana (80g)<br>1 handful of hazelnuts (15 g)	1 small sandwich of whole wheat bread (45 g) with ham (20 g) and sliced cheese (20 g)	Snack: Rice, corn and fruit with olive oil (1 portion)<br>Tangerine (150g)	1 banana (80g)<br>1 handful of almonds (15 g)
**LUNCH**	Green beans with potato (1 portion)<br>Lentils with carrot (1 portion)<br>Apple (150g)	Lettuce, corn and ham salad (1 portion)<br>Asparagus and turkey burrito (1 portion)<br>1 natural yogurt with fiber topping (10 g)	Chard with serrano ham (1 portion)<br>Cod with zucchini vichyssoise (1 portion)<br>Orange (150g)	Zucchini spaghetti (1 portion)<br>Black bean burger (1 portion)<br>1 natural yogurt with red fruit topping (10 g)	Macaroni with vegetables (1 portion)<br>Gratinéed hake in dill sauce (1 portion)<br>Pear (150g)	Steamed artichokes (1 portion)<br>Borage pancakes with mushrooms (1 portion)<br>1 natural yogurt with fruit topping (10 g)	Oatmeal paella (1 portion)<br>Flexynuggets (1 portion)<br>Orange (150g)
**AFTERNOON SNACK**	1 natural yogurt with red fruit topping (10 g)<br>Kiwi (150g)	1 small sandwich of whole wheat bread (45 g) and sliced cheese (40 g)	1 natural yogurt with fruit topping (10 g)<br>Banana (80g)	1 small sandwich of whole wheat bread (45 g) with serrano ham (40 g)	1 natural yogurt with fiber topping (10 g)<br>Strawberries (150g)	1 small toast of whole wheat bread (45 g) with tomato and fresh cheese (60 g)	1 toast of whole wheat bread (45 g) with cottage cheese nutella , cocoa and nuts (1 portion)
**DINNER**	Vegetable flan with cheese and oregano with tomato garnish (1 portion)<br>1 natural yogurt with fiber topping (10 g)	Battered hake in oatmeal popcorn and zucchini cream (1 portion)<br>Pear (150g)	Chicken wok with vegetables, curry and white rice (1 portion)<br>1 natural yogurt with fiber topping (10 g)	Baked dumplings (1 portion)<br>Apple (150g)	Cabbage omelette with peas (1 portion)<br>1 natural yogurt with red fruit topping (10 g)	Grilled pork tenderloin with french fries (1 portion)<br>Pear (150g)	Chickpea meatballs with tuna and tomato (1 portion)<br>1 natural yogurt with red fruit topping (10 g)

Week 2	DAY 1	DAY 2	DAY 3	DAY 4	DAY 5	DAY 6	DAY 7
**BREAKFAST**	1 glass of whole milk (200 ml) with 1 tablespoon of soluble cocoa (10 g)<br>1 whole wheat toast (30 g)<br>Kiwi (150g)	1 glass of whole milk (200 ml) with 1 tablespoon of soluble cocoa (10 g)<br>Breakfast cereals (30g)<br>1 banana (80g)	1 glass of whole milk (200 ml) with 1 tablespoon of soluble cocoa (10 g)<br>1 whole wheat toast (30 g)<br>Pear (150g)	1 glass of whole milk (200 ml) with 1 tablespoon of soluble cocoa (10 g)<br>Breakfast cereals (30g)<br>Apple (150g)	1 glass of whole milk (200 ml) with 1 tablespoon of soluble cocoa (10 g)<br>1 whole wheat toast (30 g)<br>Orange (150g)	1 glass of whole milk (200 ml) with 1 tablespoon of soluble cocoa (10 g)<br>Breakfast cereals (30g)<br>Kiwi (150g)	1 glass of whole milk (200 ml) with 1 tablespoon of soluble cocoa (10 g)<br>1 whole wheat toast (30 g)<br>Tangerine (150g)
**MID-MORNING SNACK**	Snack: Rice, corn and peas with olive oil (1 portion)<br>Tangerine (150g)	1 small sandwich of whole wheat bread (45 g) with ham (40 g)	1 small sandwich of whole wheat bread (45 g) with serrano ham (40 g)	Tangerine (150g)<br>1 handful of cashews (15 g)	Snack: Rice, corn and fruits with olive oil (1 portion)<br>Apple (150g)	Banana (80g)<br>1 small sandwich of whole wheat bread (30 g) and ham (20 g)	Apple (150g)<br>1 small sandwich of whole wheat bread (30 g) and ham (20 g)
**LUNCH**	Lettuce salad, carrot strips and green olives (1 portion)<br>Chickpeas, curry and spinach (1 portion)<br>1 natural yogurt with fiber topping (10 g)	Gratinéed cauliflower (1 portion)<br>Battered pomfret in tomato sauce (1 portion)<br>Apple (150g)	Tomato and fresh cheese salad (1 portion)<br>Lentils with pumpkin and chicken (1 portion)<br>1 natural yogurt with red fruit topping (10 g)	Milanese rice (1 portion)<br>Vegetable omelette (1 portion)<br>Pear (150g)	Vegetable stew (1 portion)<br>Flexiballs (1 portion)<br>1 natural yogurt with fiber topping (10 g)	Spaghetti with tomato sauce and vegetables (1 portion)<br>Baked cod with lemon (1 portion)<br>Tangerine (150g)	Zucchini cream (1 portion)<br>Flexiburger (1 portion)<br>1 natural yogurt with red fruit topping (10 g)
**AFTERNOON SNACK**	1 small sandwich of whole wheat bread (45 g) with serrano ham (40 g)	1 natural yogurt with fruit topping (10 g)<br>Strawberries (150g)	Apple (150g)<br>1 handful of walnuts (15 g)	1 small sandwich of whole wheat bread (45 g) with sliced cheese (40 g)	1 handful of hazelnuts (15 g)<br>Tangerine (150g)	1 small toast of whole wheat bread (45 g) with tomato and goat cheese (40 g)	1 glass of whole milk (150 ml)<br>Beet brownie (1 portion)
**DINNER**	Green bean, smoked salmon, sesame, walnut and fruit salad (1 portion)<br>Pear (150g)	Pork loin in apple sauce with white rice (1 portion)<br>1 natural yogurt with fiber topping (10 g)	Vegetables with pasta (1 portion)<br>Orange (150g)	Chicken schnitzel with baby carrots (1 portion)<br>1 natural yogurt with fruit topping (10 g)	Baked sweet potato stuffed with egg and melted cheese (1 portion)<br>Banana (80g)	Ham omelette with tomato sauce and vegetables (1 portion)<br>1 natural yogurt with fruit topping (10 g)	Oatmeal and pumpkin broth rice (1 portion)<br>Banana (80g)

* Daily 1 portion of whole wheat bread (30 g) at lunch and dinner.

Company products

Ready-to-eat dishes

Recipes to make at home

Supplementary Information S2: List of ingredients of the food supplied in the ALINFA diet to the study participants

1. Two mid-morning snacks based on corn and peas or fruit (GRUPO APEX: Aperitivos y Extrusionados S.A.)

- *Snack 1*: Rice, corn and peas flour and olive oil
- *Snack 2*: Rice and corn flour, banana, orange and hawthorn berry dehydrated and olive oil

2. Three types of oatmeal toppings to add to yoghurts (Harivenasa S.L.)

- *Red fruit topping*: oat flakes, pumpkin seeds, blueberries, goji berries, cherries and strawberries
- *Fiber topping*: oat flakes, pumpkin seeds, sunflower seeds, golden linseeds, brown linseeds and kiwi
- *Fruit topping:* oat flakes, sunflower seeds, pumpkin seeds, blueberries, pineapple, apple, goji berries and cherries

3. Three flexitarian products based on vegetable extrusion and meat (meatballs, nuggets and hamburgers developed by Alimentos Sanygran S.L.)

- *Flexiballs*: hydrated vegetable extruded (soy, rice and bean flour), beef, soy protein, salt, soy sauce, powder seasoning (spices), beet powder, wheat flour, sunflower oil
- *Flexinuggets*: chicken, hydrated vegetable extruded (soy, rice and bean flours), salt, seasoning (spices), tomato and beet powder
- *Flexiburger*: hydrated vegetable extruded (soy, rice and bean flour), beef, soy protein, salt, soy sauce, onion, garlic and beet powder.

4. Three precooked dishes (oatmeal paella, vegetable stew and vegetables with pasta developed by IAN S.A.U.: Navarra Food Industries).

- *Oatmeal paella*: oat, braised chicken breast, pea, sweet corn, green bean, soybeans, olive oil, red bean, sunflower seeds, onion, salt, saffron extract and soy lecithin
- *Vegetable stew:* green bean peas, artichokes, carrot, mushroom, olive oil and salt
- *Vegetables with pasta:* pasta, tomato, green bean, zucchini, onion, olive oil, green and red pepper, orange rind, sugar and salt

5. 15 ready-to-eat meals (Irigoyen Comedor Saludable S.L.):

- *Green beans with potato:* green beans, potato, olive oil, garlic and salt
- *Lentils with carrot:* lentils, tomato, onion, carrot, red and green pepper, olive oil, garlic and salt

- *Vegetable flan with cheese and oregano:* pasteurized egg, zucchini, potato, emmental cheese, carrot, red and green pepper, onion, tomato, olive oil, salt and oregano
- *Chard with serrano ham:* chard, serrano ham, olive oil, garlic and salt
- *Cod with zucchini vichyssoise:* cod, potato, zucchini, bread crumbs, butter, olive oil and salt.
- *Chicken wok with vegetables, curry and white rice:* chicken, red and green pepper, carrot, onion, potato, vinegar, olive oil and salt
- *Macaroni with vegetables:* pasta, broccoli, pumpkin, red and green pepper, olive oil and salt
- *Gratinéed hake in dill sauce:* hake, whole milk, onion, leek, pasteurized yolk, corn flour, olive oil, dill and salt
- *Cabbage omelette with peas:* cabbage, pasteurized egg, potato, peas, olive oil and salt
- *Gratinéed cauliflower:* cauliflower, wheat flour, butter, emmental cheese and white pepper and salt
- *Battered pomfret in tomato sauce:* pomfret, tomato sauce, olive oil, wheat flour, pasteurized egg, red and green pepper, onion, garlic, salt and oregano
- *Pork loin in apple sauce with white rice:* pork, onion, apple, olive oil, vinegar, ginger and salt
- *Milanese rice:* rice, carrot, mushroom, raisins, emmental cheese, olive oil, oregano and salt
- *Vegetable omelette:* pasteurized egg, red and green pepper, zucchini, carrot, olive oil and salt
- *Chicken schnitzel with baby carrots:* chicken, carrot, olive oil, garlic and salt

Table S2. Comparison of the baseline characteristics of the study participants depending on the complete or drop out of the intervention

	**Completed the intervention (n=55)**	**Abandoned the intervention (n=14)**	***p* value**
Group (ALINFA/control)	44/11	3/11	**<0.001**
Gender (boys/girls)	25/30	5/9	0.512
Age	9.07±1.73	8.92±1.59	0.778
Anthropometry			
Weight (kg)	35.12±9.72	32.47±7.79	0.299
Height (m)	1.38±0.11	1.37±0.11	0.817
BMI (kg/m^2)	18.02±3.31	16.88±1.86	0.354
Waist (cm)	61.78±8.80	60.67±6.63	0.714
SBP (mmHg)	100.66±16.59	102.67±8.15	0.963
DBP (mmHg)	67.33±10.25	65.82±2.45	0.612
Body composition			
Fat mass (kg)	8.11±4.73	7.05±2.45	0.910
Lean mass (kg)	27.01±6.06	25.42±5.75	0.238
Muscular mass (kg)	25.57±5.76	24.07±5.47	0.241
Total water (kg)	20.60±7.06	18.60±4.21	0.172
Questionnaires			
Quality of life (KINDL)	88.43±6.67	89.58±5.81	0.612
Physical activity (PAQ-C)	3.08±0.60	2.95±0.64	0.734
**Diet quality (Kidmed index)**	7.09±2.22	6.33±1.43	0.263
Interpretation:<br>-Low diet quality<br>-Need to improve dietary pattern<br>-Optimal MD	4 (7.27%)<br>28 (50.91%)<br>23 (41.82%)	2 (14.29%)<br>8 (57.14%)<br>4 (28.57%)	0.545
Biochemistry			
Glucose (mg/dl)	92.85 ± 6.17	94.18 ± 2.38	0.537
Insulin (µIU/ml)	9.68 ± 4.66	8.12 ± 3.48	0.272
Total cholesterol (mg/dl)	173.76 ± 26.72	188.0 ± 36.16	0.128
HDL-c (mg/dl)	62.32 ± 10.66	64.20 ± 11.65	0.588
LDL-c (mg/dl)	100.33 ± 24.31	112.96 ± 27.64	0.117
TNF-α (pg/ml)	5.24 ± 1.35	5.83 ± 1.41	0.192
Leptin (ng/ml)	1.72 ± 1.5	1.11 ± 0.47	0.669
CRP (mg/dl)	0.97 ± 1.24	0.63 ± 0.40	0.962

Table S3. Change in consumption (g/day) of the main food groups after the intervention in ALINFA study.

	Control (n=11)			ALINFA (n=44)			Change between groups (*p* value) [b]
	**Pre-intervention**	**Post-intervention**	***p* value [a]**	**Pre-intervention**	**Post-intervention**	***p* value [a]**	
Whole dairy (g/day)	419.4 ± 281.3	367.0 ± 227.7	0.521	356.1 ± 284.6	337.15 ± 235.9	0.514	0.776
Low-free fat dairy (g/day)	181.2 ± 228.0	122.0 ± 188.1	0.917	104.1 ± 131.1	105.07 ± 190.0	0.732	0.901
Egg (g/day)	19.48 ± 5.98	24.28 ± 9.45	0,437	20.88 ± 8.81	20.26 ± 7.92	0.806	0.412
Lean meat (g/day)	95.10 ± 39.85	121.18 ± 49.04	0,437	100.5 ± 36.02	110.4 ± 48.41	0.308	0.772
Fatty meat (g/day)	36.26 ± 19.03	45.66 ± 32.99	0,437	**37.61 ± 19.65**	**25.83 ± 27.34**	**0.014**	0.097
White fish (g/day)	18.88 ± 11.19	19.50 ± 13.44	0,911	**14.72 ± 8.10**	**21.00 ± 8.76**	**0.001**	0.382
Fatty fish (g/day)	13.44 ± 12.82	12.87 ± 13.03	0,437	13.84 ± 10.66	14.25 ± 11.13	0.772	0.817
Vegetable (g/day)	301.7 ± 168.1	326.7 ± 167.4	0,774	234.1 ± 109.6	226.3 ± 84.7	0.847	0.776
Fruit (g/day)	372.4 ± 322.0	534.5 ± 299.3	0,437	328.5 ± 216.1	320.9 ± 142.2	0.260	0.190
Pulse (g/day)	30.79 ± 36.84	27.49 ± 10.17	0,437	**27.01 ± 11.29**	**33.19 ± 16.15**	**0.004**	0.901
Refined grains (g/day)	135.1 ± 68.44	148.7 ± 85.59	0,774	**105.0 ± 43.06**	**81.8 ± 36.37**	**0.008**	0.259
Whole grains (g/day)	8.66 ± 13.02	14.59 ± 15.88	0,521	**3.13 ± 8.08**	**46.72 ± 50.55**	**<0.001**	0.082
Nut (g/day)	3.62 ± 4.79	6.73 ± 9.43	0,774	**6.08 ± 6.86**	**12.29 ± 7.95**	**<0.001**	0.473
Olive oil (g/day)	27.65 ± 15.45	28.63 ± 11.85	0,750	23.38 ± 9.53	21.79 ± 7.83	0.741	0.776
Other fats (g/day)	21.30 ± 37.60	25.27 ± 26.28	0,769	14.93 ± 20.44	9.08 ± 11.26	0.195	0.190
Pastries/ confectionery (g/day)	24.52 ± 11.32	18.11 ± 16.51	0,437	**25.08 ± 12.75**	**13.03 ± 12.87**	**<0.001**	0.744
Fast food (g/day)	45.64 ± 2.85	47.90 ± 21.48	0,917	**56.72 ± 20.44**	**39.44 ± 19.96**	**<0.001**	0.097
Sugars (g/day)	75.09 ± 50.30	139.3 ± 266.1	0,774	**79.76 ± 64.44**	**51.29 ± 59.58**	**0.001**	0.423
Sweetened foods (g/day)	11.09 ± 13.22	33.78 ± 71.78	0,437	7.17 ± 19.92	3.93 ± 8.76	0.104	0.097
Water (g/day)	970.9 ± 704.1	908.7 ± 322.3	0,854	873.0 ± 579.6	885.9 ± 490.9	0.474	0.776
Salt (g/day)	4.72 ± 3.40	3.46 ± 1.83	0,774	3.01 ± 1.89	2.80 ± 1.67	0.238	0.901

Data are mean ± SD. Benjamini-Hochberg adjustment was applied [#] Significant differences between groups at baseline. [a] p values based on Student's t-test or Wilcoxon test. Statistical significance defined as $p < 0.05$. [b] p values based on Student's t-test or Mann-Whitney U. Statistical significance defined as $p < 0.05$.
